# Psychiatric adverse events in three phase III trials of eslicarbazepine acetate for focal seizures

**DOI:** 10.1002/epi4.12635

**Published:** 2022-08-30

**Authors:** Hamada Altalib, Todd Grinnell, David Cantu, Fábio Ikedo, Mariana Vieira, Yi Zhang, David Blum

**Affiliations:** ^1^ Department of Neurology Yale School of Medicine New Haven Connecticut USA; ^2^ Sunovion Pharmaceuticals Inc. Marlborough Massachusetts USA; ^3^ Sunovion Pharmaceuticals Inc. Fort Lee New Jersey USA; ^4^ Pharmacovigilance Compliance BIAL – Portela & Cª, S.A. São Mamede do Coronado Portugal

**Keywords:** anxiety, depression, eslicarbazepine acetate, focal seizures, psychiatric, psychotropic

## Abstract

**Objective:**

Eslicarbazepine acetate (ESL) is a once‐daily (QD), oral anti‐seizure medication for the treatment of focal (partial‐onset) seizures. Here, we evaluate risk factors for the development of psychiatric treatment‐emergent adverse events (TEAEs) in clinical trials of adjunctive ESL in adults with focal seizures.

**Methods:**

This post‐hoc analysis evaluated data pooled from three Phase III, randomized, double‐blind, placebo‐controlled trials (BIA‐2093‐301, ‐302, ‐304). After an 8‐week baseline period, patients were randomized equally to receive placebo, ESL 400 mg (not reported here), 800 mg, or 1200 mg QD (up to 2‐week titration; 12‐week maintenance; optional open‐label extension [OLE]). Incidences of psychiatric TEAEs were evaluated according to three separate criteria: medical history of psychiatric disorders (yes/no); baseline use of psychotropic drugs (yes/no); Montgomery–Åsberg Depression Rating Scale (MADRS) score at baseline (0–6: normal; 7–19: mild depression; 20–34: moderate depression).

**Results:**

The analysis populations comprised 1251 patients for the controlled study period and 1137 patients for the 1‐year OLE. Psychiatric TEAE incidence was similar between patients taking ESL and placebo in the controlled and OLE study periods and was not related to ESL dose. Psychiatric TEAEs generally occurred more frequently in patients with a medical history of psychiatric disorders, using psychotropic drugs, or with depressive symptoms than in those without a history, not using psychotropic drugs, or with no depressive symptoms. Depression and anxiety were the most frequently reported psychiatric TEAEs.

**Significance:**

Overall, in clinical trials of ESL in adults with focal seizures, incidences of psychiatric events were not statistically different between patients taking ESL and placebo, were not related to ESL dose, and generally occurred more frequently in patients with baseline psychiatric symptoms or a history of psychiatric disorders. Long‐term exposure to ESL was not associated with a marked increase in the incidence of psychiatric TEAEs.

**Key Points**

- Psychiatric TEAE incidence did not differ statistically with ESL vs placebo.
- Patients with a psychiatric history experienced more psychiatric TEAEs.
- Patients with higher MADRS scores at baseline experienced more psychiatric TEAEs.
- Depression and anxiety were the most frequently reported psychiatric TEAEs.

## 1 INTRODUCTION

Psychiatric conditions are common comorbidities in patients with epilepsy, possibly due to an underlying pathology involved in the causation of both seizures and psychiatric/behavioral disorders.^1^, ^2^, ^3^ One in three epilepsy patients is likely to experience a psychiatric condition during his/her lifetime, with depression and anxiety disorders most common.^3^, ^4^, ^5^ In addition, many anti‐seizure medications (ASMs) carry a risk of psychiatric adverse events (AEs) such as depression, anxiety, hyperactivity, agitation, irritability, aggression, or psychosis.^2^, ^6^, ^7^ Conversely, some ASMs (including some voltage‐gated sodium channel inhibitors) have demonstrated mood‐stabilizing and/or anxiolytic properties.^2^, ^3^, ^8^ Mood and anxiety disorders increase the risk of morbidity and premature mortality and are strong predictors of a poor quality of life in patients with epilepsy, often having a more severe impact on quality of life than seizures themselves^3^, ^5^, ^8^; therefore, anticipating, detecting, and treating psychiatric conditions is an important aspect of care for patients with epilepsy.

Eslicarbazepine acetate (ESL) is a once‐daily (QD) oral ASM for the treatment of focal (partial‐onset) seizures.^9^, ^10^ Data from three randomized, double‐blind, Phase III, placebo‐controlled studies (BIA‐2093‐301, ‐302, and ‐304) demonstrated that ESL was effective and generally well tolerated as an adjunctive therapy in adults with treatment‐refractory focal seizures.^11^, ^12^, ^13^, ^14^ Psychiatric treatment‐emergent AEs (TEAEs) were reported infrequently during these studies; however, depression and suicidality‐related TEAEs were reported more frequently in patients taking ESL than in the placebo group.^15^ Therefore, a more detailed analysis of psychiatric TEAEs during long‐term treatment with ESL is warranted.

Previous work has identified that a history of mood disorders can be associated with an increased risk of developing psychiatric TEAEs with use of ASMs.^3^, ^16^ We therefore conducted this post–hoc analysis to identify risk factors for the development of psychiatric TEAEs in clinical trials of adjunctive ESL in adults with focal seizures. We evaluated the occurrence of psychiatric TEAEs according to the presence or absence of a medical history (documented diagnosis) of psychiatric disorders, baseline use of psychotropic drugs, and baseline Montgomery–Åsberg Depression Rating Scale (MADRS) score category, to identify whether any of these patient characteristics were associated with the likelihood of experiencing psychiatric TEAEs.

## 2 METHODS

### 2.1 Standard protocol approvals, registration, and patient consent

This post–hoc analysis evaluated data pooled from three Phase III, randomized, double‐blind, placebo‐controlled trials of adjunctive ESL to determine psychiatric outcomes in patients taking ESL vs placebo. The three studies (BIA‐2093‐301 [NCT00957684]; ‐302 [NCT00957047]; and ‐304 [NCT00988429]; registered at ClinicalTrials.gov) were conducted at centers across 35 countries between 2004 and 2012, in accordance with the principles of the Declaration of Helsinki, the International Conference on Harmonization guidelines, and all national, state, and local laws of the pertinent regulatory authorities. Relevant independent ethics committees/institutional review boards provided approval, and all patients provided written informed consent.

### 2.2 Study design

The study designs (including sample size determination, eligibility criteria, and details of randomization and blinding) of Studies 301, 302, and 304 have been reported previously.^11^, ^12^, ^13^, ^14^ For Part 1, after an 8‐week baseline period, patients were randomized equally to receive placebo, ESL 400 mg (Studies 301/302 only; not reported here), 800 mg, or 1200 mg QD for a 2‐week titration period, and a 12‐week maintenance period. In Study 301, patients titrated from ESL 400 mg in Week 1 to 800 mg in Week 2 to the maintenance dose (ESL 800 mg or 1200 mg) in Week 3. In Study 302, patients initiated treatment with ESL 800 mg in Weeks 1 and 2, then either maintained treatment with ESL 800 mg, or titrated to the maintenance dose of 1200 mg in Week 3. In Study 304, patients titrated from ESL 400 mg in Weeks 1 and 2 to a maintenance dose of 800 mg, or from ESL 800 mg in Weeks 1 and 2 to a maintenance dose of 1200 mg. Patients continued to receive stable doses of concomitant ASMs during the studies. At the end of the 12‐week maintenance period, all patients regardless of dose, which was not unblinded at the time of transition, had the option to continue into an open‐label extension (OLE) study or to discontinue treatment with ESL. For those discontinuing ESL, patients in Studies 301 and 304 entered a 4‐ or 2‐week tapering‐off period, respectively, and patients in Study 302 immediately discontinued treatment. In Study 301, patients were required to complete the 4‐week tapering‐off period even if continuing into the OLE.

Patients who entered the OLE (Part 2) received a starting dose of ESL 800 mg QD for 1 month. Thereafter, investigators could increase the ESL dose to address seizures or decrease the dose to address AEs. The maximum and minimum allowed daily ESL doses were 1200 mg (Studies 301 and 302) or 1600 mg (Study 304) and 400 mg QD, respectively.

### 2.3 Patients

Patients aged ≥16 (Study 304) or ≥18 (Studies 301 and 302) years with refractory focal seizures during stable treatment with 1–2 ASMs (Studies 301 and 304) or 1–3 ASMs (Study 302) were eligible for inclusion. Patients were required to have had ≥4 focal seizures during each of the two 4‐week baseline periods, with no seizure‐free period ˃21 consecutive days (Studies 301 and 302) or ≥8 focal seizures during baseline, with ≥3 seizures in each 4‐week period and no seizure‐free period ˃28 consecutive days (Study 304). Further details of the inclusion and exclusion criteria have been reported previously.^11^, ^12^, ^13^, ^14^ Patients taking oxcarbazepine, or with a history of suicide attempts or major psychiatric disorders, including schizophrenia, were excluded from participation.

### 2.4 Assessment and analysis of TEAEs

Individual patient data from Studies 301, 302, and 304 were pooled and analyzed. TEAEs were reported by investigators during the studies and coded using the Medical Dictionary for Regulatory Activities Version 13.1. TEAE data were collected at Day 0, Week 2 (end of the titration period), Week 8, Week 14, Week 18, and Week 22 (Study 301 only), and then approximately every 12 weeks during Part 2 (1‐year OLE). Additional events were identified by clinical audit of investigator records and case report forms, and by review of patient narratives and serious AE reports (Council for International Organizations of Medical Sciences forms). Signs and symptoms relating to any reported diagnoses were recorded as additional TEAEs. Psychiatric TEAEs were identified as those within the System Organ Class “Psychiatric Disorders” and were not necessarily supported by a clinical diagnosis.

Patients with psychiatric TEAEs were categorized and evaluated according to psychiatric history or current psychiatric symptom status based on three separate criteria: (1) medical history of psychiatric disorders based on questions asked during screening: yes/no; (2) baseline use of psychotropic drugs: yes/no; (3) categorical MADRS score (indicating presence of depressive symptoms) at baseline: 0–6 = normal, 7–19 = mild depression, 20–34 = moderate depression. No psychiatric TEAEs were reported in the five patients who had baseline MADRS scores >34 (severe depression); therefore, outcome data for this group are not reported in the tables. Numbers and percentages of patients with psychiatric TEAEs occurring in >1% of the total ESL population (and in >1 patient) were reported for each subgroup. Kaplan–Meier analyses evaluated time to first TEAE according to history of psychiatric disorders, baseline use of psychotropic drugs, or categorical MADRS score.

The analysis population included all patients who received ≥1 dose of the study drug. Data were not reported for the ESL 400 mg treatment group, as this dose was not effective in reducing focal seizure frequency.^14^ *P*‐values for differences between incidences of psychiatric TEAEs were calculated post–hoc, using Chi‐square tests when the minimum expected cell value was ≥5, and Fisher's exact tests when the minimum expected cell value was <5. Chi‐square or Fisher's exact tests were applied to incidence rates without correcting for multiplicity or the post–hoc nature of the hypotheses. Hazard ratios (HRs) with corresponding 95% confidence intervals (CIs) and *P*‐values were also estimated for each of the three psychiatric criteria using a non‐stratified Cox proportional hazards model. There were two comparison groups for medical history of psychiatric disorders and baseline use of psychotropic drugs (yes vs no), and three comparison groups for categorical MADRS score at baseline (0–6 vs 7–19, 0–6 vs 20–34, and 7–19 vs 20–34).

## 3 RESULTS

### 3.1 Patients

The analysis population for the double‐blind, placebo‐controlled study period (Part 1) comprised 1251 patients (placebo: n = 426; ESL 800 mg: n = 415; ESL 1200 mg: n = 410). The analysis population for the 1‐year OLE period (Part 2) comprised 1137 patients taking ESL.

### 3.2 Medical history of psychiatric disorders

Baseline demographics and clinical characteristics according to medical history of psychiatric disorders are shown in Table 1A. During both the controlled and OLE study periods, patients with a psychiatric history were slightly older than those with no such history. In addition, during both the controlled and OLE study periods, use of lamotrigine (LTG) or levetiracetam (LEV) was more frequent in patients with a psychiatric history, whereas use of carbamazepine (CBZ) or valproic acid (VPA) was more frequent in patients without a psychiatric history. Approximately 40% of patients with a psychiatric history were using a psychotropic medication at baseline, and the most commonly used psychotropic drugs were citalopram and fluoxetine.

**TABLE 1:** (A) Baseline demographics and clinical characteristics and (B) incidences of psychiatric TEAEs,^a^ during the double‐blind, placebo‐controlled study period and OLE, according to history of psychiatric disorders

(A)						
Controlled study period						
	Placebo		ESL 800 mg		ESL 1200 mg	
	Medical history of psychiatric disorders					
	Yes	No	Yes	No	Yes	No
	n = 81	n = 345	n = 103	n = 312	n = 94	n = 316
Age, years, median (range)	43.0 (16–66)	36.0 (16–68)	42.0 (16–75)	37.0 (16–71)	38.5 (16–69)	36.0 (16–69)
Duration of epilepsy, years, mean (SD)	21.8 (13.8)	21.4 (13.9)	23.8 (13.5)	21.2 (12.4)	22.1(12.8)	20.7 (12.6)
ASMs, n (%)						
1	20 (24.7)	96 (27.9)	21 (20.6)	86 (27.7)	28 (29.8)	89 (28.3)
2	60 (74.1)	237 (68.9)	76 (74.5)	216 (69.7)	59 (62.8)	221 (70.2)
≥3	1 (1.2)	11 (3.2)	5 (4.9)	8 (2.6)	7 (7.4)	5 (1.6)
ASMs,^b^ n (%)						
Carbamazepine	21 (25.9)	177 (51.3)	36 (35.0)	168 (53.8)	30 (31.9)	174 (55.1)
Lamotrigine	25 (30.9)	83 (24.1)	29 (28.2)	64 (20.5)	29 (30.9)	76 (24.1)
Valproic acid	12 (14.8)	83 (24.1)	18 (17.5)	77 (24.7)	15 (16.0)	71 (22.5)
Levetiracetam	19 (23.5)	70 (20.3)	28 (27.2)	52 (16.7)	22 (23.4)	51 (16.1)
Use of any psychotropic medication,^c^ n (%)	29 (35.8)	5 (1.4)	43 (41.7)	5 (1.6)	34 (36.2)	7 (2.2)
SSRI	18 (22.2)	0 (0.0)	30 (29.1)	1 (0.3)	23 (24.5)	0 (0.0)
Citalopram	3 (3.7)	0 (0.0)	11 (10.7)	0 (0.0)	8 (8.5)	0 (0.0)
Escitalopram	4 (4.9)	0 (0.0)	5 (4.9)	0 (0.0)	5 (5.3)	0 (0.0)
Fluoxetine	7 (8.6)	0 (0.0)	4 (3.9)	0 (0.0)	7 (7.4)	0 (0.0)
Sertraline	3 (3.7)	0 (0.0)	9 (8.7)	0 (0.0)	4 (4.3)	0 (0.0)
MADRS score, median	8.0	6.0	8.0	6.0	8.5	8.0

OLE		
	OL ESL	
	Medical history of psychiatric disorders	
	Yes	No
	n = 242	n = 895
Age, years, median (range)	40.0 (16–75)	35.0 (16–71)
Duration of epilepsy, years, mean (SD)	22.7 (12.9)	20.9 (13.0)
ASMs, n (%)		
1	60 (24.8)	248 (27.8)
2	170 (70.2)	622 (69.7)
≥3	12 (5.0)	23 (2.6)
ASMs,^b^ n (%)		
Carbamazepine	69 (28.5)	477 (53.3)
Lamotrigine	76 (31.4)	206 (23.0)
Valproic acid	45 (18.6)	215 (24.0)
Levetiracetam	61 (25.2)	166 (18.5)
Use of any psychotropic medication,^c^ n (%)	96 (39.7)	19 (2.1)
SSRI	61 (25.2)	1 (0.1)
Citalopram	20 (8.3)	0 (0.0)
Escitalopram	13 (5.4)	0 (0.0)
Fluoxetine	15 (6.2)	0 (0.0)
Sertraline	13 (5.4)	0 (0.0)
MADRS score, median	9.0	7.0

(B)						
Controlled study period						
TEAE, n (%)	Placebo		ESL 800 mg		ESL 1200 mg	
	Medical history of psychiatric disorders					
	Yes	No	Yes	No	Yes	No
	n = 81	n = 345	n = 103	n = 312	n = 94	n = 316
Any psychiatric TEAE	14 (17.3)	18 (5.2)	10 (9.7)	22 (7.1)	12 (12.8)	17 (5.4)
	***P* = 0.0002** ^**d**^		*P* = 0.3807^d^		***P* = 0.0142** ^**d**^	
	–		*P* = 0.1299^e^	–	*P* = 0.4021^e^	–
Anxiety	4 (4.9)	5 (1.4)	4 (3.9)	5 (1.6)	1 (1.1)	0
	***P* = 0.0494** ^**d**^		*P* = 0.2606^d^		*P* = 0.0664^d^	
	–		*P* = 0.7276^e^	–	*P* = 0.1250^e^	–
Depression	4 (4.9)	3 (0.9)	2 (1.9)	4 (1.3)	6 (6.4)	3 (0.9)
	***P* =0.0095** ^**d**^		*P* = 0.6267^d^		***P* = 0.0016** ^**d**^	
	–		*P* = 0.2559^e^	–	*P* = 0.6814^e^	–
Insomnia	0	3 (0.9)	2 (1.9)	6 (1.9)	3 (3.2)	2 (0.6)

OLE		
TEAE, n (%)	OL ESL	
	Medical history of psychiatric disorders	
	Yes	No
	n = 242	n = 895
Any psychiatric TEAE	38 (15.7)	80 (8.9)
	***P* = 0.0022** ^a^	
Aggression	3 (1.2)	4 (0.4)
Anxiety	13 (5.4)	13 (1.5)
	***P* = 0.0003** ^a^	
Confusional state	4 (1.7)	4 (0.4)
Depression	10 (4.1)	14 (1.6)
	***P* = 0.0137** ^a^	
Insomnia	5 (2.1)	13 (1.5)

*Note:* Individual psychiatric TEAEs occurring in >1% of the total ESL population in either subgroup are reported. For the controlled study period, the total ESL population included the 800 and 1200 mg doses, as well as a 400 mg dose group (data not reported here). *P*‐values <0.05 are in bold.

Abbreviations: ASM, anti‐seizure medication; ESL, eslicarbazepine acetate; MADRS, Montgomery–Åsberg Depression Rating Scale; OL, open‐label; OLE, open‐label extension; SD, standard deviation; SSRI, selective serotonin reuptake inhibitor; TEAE, treatment‐emergent adverse event.

^a^ Yes vs no.

^b^ Psychiatric TEAEs are investigator‐reported Medical Dictionary for Regulatory Activities preferred terms and not clinical diagnoses.

^c^ Used by >15% of patients.

^d^ Used by >5% of patients.

^e^ Patients with a medical history of psychiatric disorders in the ESL vs placebo group.

**FIGURE 1:**
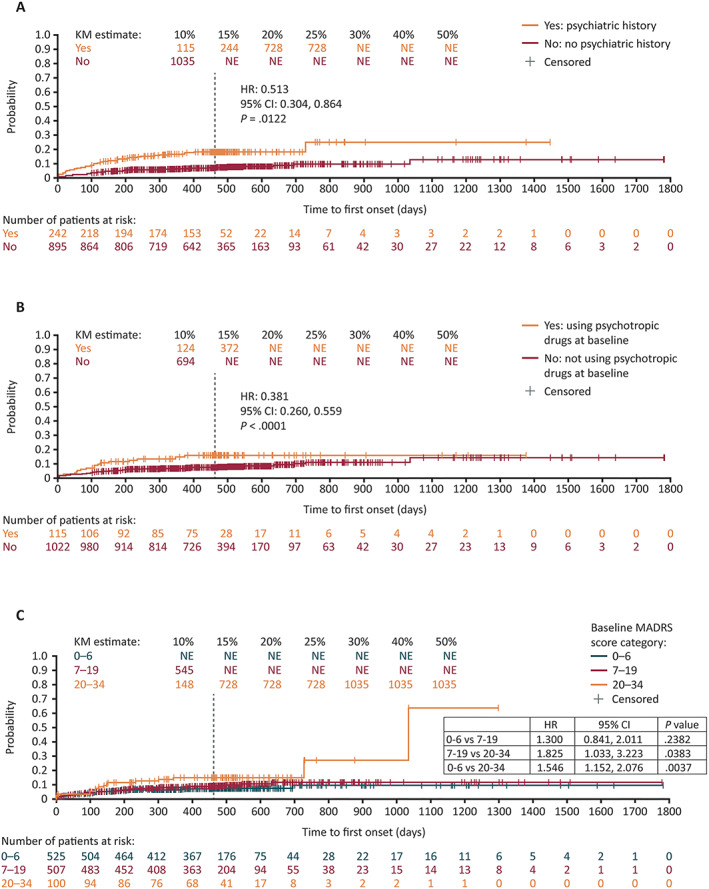
Time to first psychiatric TEAE during the controlled and 1‐year OLE study periods, according to: (A) history of psychiatric disorders; (B) baseline use of psychotropic drugs; (C) baseline MADRS total score. Individual psychiatric TEAEs occurring in >1% of patients in either subgroup during the 1‐year OLE are reported. The vertical dashed line indicates the end of the 1‐year OLE. For patients who discontinued prior to completing the 1‐year OLE, their last contact date is reported as the censoring date. For patients who were ongoing at the completion of the 1‐year OLE, their end date was reported as the censoring date. CI, confidence interval; HR, hazard ratio; KM, Kaplan–Meier; MADRS, Montgomery–Åsberg Depression Rating Scale; NE, not evaluable; OLE, open‐label extension; TEAE, treatment‐emergent adverse event

During the controlled study period, in the placebo and ESL 1200 mg groups, the overall incidence of psychiatric TEAEs and the incidence of depression were both higher in patients with a psychiatric history than in those with no such history (Table 1B). However, there was no clear relationship between the presence/absence of a psychiatric history and incidence of insomnia (the remaining psychiatric TEAE that occurred in >1% of patients taking ESL). It is of note that TEAE reports were not necessarily accompanied by a clinical diagnosis. A Kaplan–Meier analysis of time to first psychiatric TEAE confirmed that psychiatric TEAEs occurred earlier and in a higher proportion of patients with (vs without) a psychiatric history during the controlled and 1‐year OLE study periods (HR: 0.381 [95% CI: 0.26, 0.56]; *P* < 0.0001) (Figure 1A). Overall, in patients with a psychiatric history, there was no clear difference in the incidence of psychiatric TEAEs between those taking ESL (either dose) and placebo.

During the 1‐year OLE, consistent with the controlled study period, the overall incidence of psychiatric TEAEs and incidences of anxiety and depression were higher in patients who had a psychiatric history than in those with no such history (Table 1B). In addition, aggression, confusional state, and insomnia were reported in >1%, but less than 2.5% of patients taking ESL in either subgroup during the OLE.

### 3.3 Baseline use of psychotropic drugs

Baseline demographics and clinical characteristics according to baseline use of psychotropic drugs are shown in Table 2A. During both study periods, patients using psychotropic drugs at baseline were slightly older than those who were not. In addition, baseline use of LTG or LEV was generally more frequent in patients using psychotropic drugs at baseline, whereas use of CBZ or VPA was more frequent in those who were not (OL ESL: LTG yes 33% vs no 24%, LEV yes 29% vs no 19%, CBZ yes 28% vs no 50%, VPA yes 18% vs no 23%). The most commonly used psychotropic drugs were citalopram and fluoxetine, and overall selective serotonin reuptake inhibitors were the most commonly used psychotropic drugs (by >50% of patients) in the “yes” subgroup. Approximately 15% of patients not using psychotropic drugs at baseline had a medical history of psychiatric disorders.

**TABLE 2:** (A) Baseline demographics and clinical characteristics and (B) incidences of psychiatric TEAEs^a^, during the double‐blind, placebo‐controlled study period and OLE, according to baseline use of psychotropic drugs

(A)						
Controlled study period						
TEAE, n (%)	Placebo		ESL 800 mg		ESL 1200 mg	
	Baseline use of psychotropic drugs					
	Yes	No	Yes	No	Yes	No
	n = 34	n = 392	n = 48	n = 367	n = 41	n = 369
Age, years, median (range)	42.5 (16–64)	36.0 (16–68)	43.5 (16–75)	37.0 (16–71)	38.0 (17–60)	36.0 (16–69)
Duration of epilepsy, years, mean (SD)	19.3 (12.7)	21.7 (13.9)	25.0 (14.7)	21.4 (12.4)	19.4 (12.3)	21.2 (12.7)
ASMs, n (%)						
1	4 (11.8)	112 (28.6)	9 (18.8)	98 (26.9)	17 (41.5)	100 (27.2)
2	30 (88.2)	267 (68.3)	38 (79.2)	254 (69.8)	23 (56.1)	257 (69.8)
≥3	0 (0.0)	12 (3.1)	1 (2.1)	12 (3.3)	1 (2.4)	11 (3.0)
ASMs,^b^ n (%)						
Carbamazepine	10 (29.4)	188 (48.0)	17 (35.4)	187 (51.0)	12 (29.3)	192 (52.0)
Lamotrigine	9 (26.5)	99 (25.3)	14 (29.2)	79 (21.5)	14 (34.1)	91 (24.7)
Valproic acid	4 (11.8)	91 (23.2)	6 (12.5)	89 (24.3)	8 (19.5)	78 (21.1)
Levetiracetam	10 (29.4)	79 (20.2)	17 (35.4)	63 (17.2)	6 (14.6)	67 (18.2)
Psychotropic medications used,^c^ n (%)						
SSRI	18 (52.9)	–	31 (64.6)	–	23 (56.1)	–
Amitriptyline	6 (17.6)	–	3 (6.3)	–	6 (14.6)	–
Citalopram	3 (8.8)	–	11 (22.9)	–	8 (19.5)	–
Duloxetine	2 (5.9)	–	2 (4.2)	–	0 (0.0)	–
Escitalopram	4 (11.8)	–	5 (10.4)	–	5 (12.2)	–
Fluoxetine	7 (20.6)	–	4 (8.3)	–	7 (17.1)	–
Paroxetine	2 (5.9)	–	5 (10.4)	–	1 (2.4)	–
Piracetam	3 (8.8)	–	4 (8.3)	–	3 (7.3)	–
Sertraline	3 (8.8)	–	9 (18.8)	–	4 (9.8)	–
Trazodone	2 (5.9)	–	2 (4.2)	–	3 (7.3)	–
Venlafaxine	0 (0.0)	–	1 (2.1)	–	3 (7.3)	–
Medical history of psychiatric disorders, n (%)	29 (85.3)	52 (13.3)	43 (89.6)	60 (16.3)	34 (82.9)	60 (16.3)
MADRS score	8.0	7.0	8.0	6.0	10.0	7.0

OLE		
	OL ESL	
	Baseline use of psychotropic drugs	
	Yes	No
	n = 115	n = 1022
Age, years, median (range)	42.0 (16–75)	36.0 (16–71)
Duration of epilepsy, years, mean (SD)	20.9 (13.0)	21.3 (12.9)
ASMs, n (%)		
1	25 (21.7)	283 (27.7)
2	88 (76.5)	704 (69.0)
≥3	2 (1.7)	33 (3.2)
ASMs,^b^ n (%)		
Carbamazepine	32 (27.8)	514 (50.3)
Lamotrigine	38 (33.0)	244 (23.9)
Valproic acid	21 (18.3)	239 (23.4)
Levetiracetam	33 (28.7)	194 (19.0)
Psychotropic medications used,^c^ n (%)		
SSRI	62 (53.9)	–
Amitriptyline	12 (10.4)	–
Citalopram	20 (17.4)	–
Escitalopram	13 (11.3)	–
Fluoxetine	15 (13.0)	–
Paroxetine	6 (5.2)	–
Piracetam	14 (12.2)	–
Sertraline	13 (11.3)	–
Trazodone	7 (6.1)	–
Medical history of psychiatric disorders, n (%)	96 (83.5)	146 (14.3)
MADRS score, median	10.0	7.0

(B)						
Controlled study period						
TEAE, n (%)	Placebo		ESL 800 mg		ESL 1200 mg	
	Baseline use of psychotropic drugs					
	Yes	No	Yes	No	Yes	No
	n = 32	n = 394	n = 53	n = 362	n = 38	n = 372
Any psychiatric TEAE	6 (18.8)	26 (6.6)	8 (15.1)	24 (6.6)	8 (21.1)	21 (5.6)
	***P* = 0.0121** ^**d**^		***P* = 0.0310** ^**d**^		***P* = 0.0004** ^**d**^	
	–		*P* = 0.6598^e^	–	*P* = 0.8104^e^	–
Anxiety	1 (3.1)	8 (2.0)	3 (5.7)	6 (1.7)	0	1 (0.3)
	*P* = 0.6788^d^		*P* = 0.0617^d^		*P* = 0.7490^d^	
	–		*P* = 0.5928^e^	–	*P* = 0.2724^e^	–
Depressed mood	0	4 (1.0)	0	2 (0.6)	1 (2.6)	0
Depression	3 (9.4)	4 (1.0)	4 (7.5)	2 (0.6)	6 (15.8)	3 (0.8)
	***P* = 0.0003** ^**d**^		***P* < 0.0001** ^**d**^		***P* < 0.0001** ^**d**^	
	–		*P* = 0.7665^e^	–	*P* = 0.4245^e^	–
Insomnia	0	3 (0.8)	1 (1.9)	7 (1.9)	0	5 (1.3)
Nightmare	0	0	1 (1.9)	0	1 (2.6)	0

OLE		
TEAE, n (%)	OL ESL	
	Baseline use of psychotropic drugs	
	Yes	No
	n = 115	n = 1022
Any psychiatric TEAE	15 (13.0)	103 (10.1)
	*P* = 0.3229^a^	
Anxiety	7 (6.1)	19 (1.9)
	***P* = 00116** ^a^	
Depression	5 (4.3)	19 (1.9)
	*P* = 0.0862^a^	
Insomnia	2 (1.7)	16 (1.6)

*Note:* Individual psychiatric TEAEs occurring in >1% of the total ESL population in either subgroup are reported. For the controlled study period, the total ESL population included the 800 mg and 1200 mg doses, as well as a 400 mg dose group (data not reported here). *P*‐values <0.05 are in bold.

Abbreviations: ASM, anti‐seizure medication; ESL, eslicarbazepine acetate; MADRS, Montgomery–Åsberg Depression Rating Scale; OL, open‐label; OLE, open‐label extension; SD, standard deviation; SSRI, selective serotonin reuptake inhibitor; TEAE, treatment‐emergent adverse event.

^a^ Yes vs no.

^b^ Psychiatric TEAEs are investigator‐reported Medical Dictionary for Regulatory Activities preferred terms and not clinical diagnoses.

^c^ Used by >15% of patients.

^d^ Used by >5% of patients.

^e^ Patients with a medical history of psychiatric disorders in the ESL vs placebo group.

During the controlled study period, the overall incidence of psychiatric TEAEs, as well as the incidence of depression, were higher in patients using psychotropic drugs at baseline than in those not using these drugs, in all treatment groups (Table 2B). Odds ratios in those using psychotropic drugs for the overall incidence of psychiatric TEAEs were 1.7 (0.6, 5.3) in the placebo group, 1.1 (0.4, 3.3) in the ESL 800 mg group, and 3.3 (1.3, 8.2) in the ESL 1200 mg group. For the incidence of depression, odds ratios were 2.0 (0.2, 16.7) in the placebo group, 1.5 (0.2, 13.5) in the ESL 800 mg group, and 20.9 (5.0, 87.3) in the ESL 1200 mg group. However, there was no clear relationship between baseline use of psychotropic drugs and incidences of anxiety, depressed mood, insomnia, or nightmare. Note that TEAEs were analyzed according to the verbatim terms provided by investigators and were not grouped into categories (e.g., investigator reports of depression and depressed mood remained separate for safety analysis). Furthermore, in patients using psychotropic drugs at baseline, there was no clear difference in psychiatric TEAE incidence between those taking ESL (either dose) and placebo. A Kaplan–Meier analysis of time to first psychiatric TEAE confirmed that psychiatric TEAEs occurred earlier and in a higher proportion of patients using (vs not using) psychotropic drugs during the controlled and 1‐year OLE study periods (HR: 0.513 [95% CI: 0.30, 0.86]; *P* = 0.0122) (Figure 1B).

During the OLE, there was no clear difference in overall incidence of psychiatric TEAEs between patients using psychotropic drugs at baseline and those who were not (Table 2B). However, the incidence of anxiety was higher in patients using psychotropic drugs at baseline than in those not using these drugs (odds ratio: 3.4 [1.4, 8.3]).

### 3.4 Baseline MADRS total score

Baseline demographics and clinical characteristics according to MADRS total score are shown in Table 3A. The most commonly used psychotropic drugs were amitriptyline and citalopram and psychotropic medication use at baseline was most frequent in patients with moderate depression (MADRS total score: 20–34; placebo: 11%; ESL 800 mg: 18%; ESL 1200 mg: 21%; OL ESL: 21%).Furthermore, the proportions of patients with a medical history of psychiatric disorders increased with increasing MADRS total score in the OLE (ESL: 0–6, 18%; 7–19, 24%; 20–34, 28%).

**TABLE 3:** (A) Baseline demographics and clinical characteristics and (B) incidences of psychiatric TEAEs^a^, during the double‐blind, placebo‐controlled study period and OLE, according to baseline MADRS total score.

Controlled study period									
	Placebo		ESL 800 mg			ESL 1200 mg			
	Baseline MADRS total score								
	0–6	7–19	20–34	0–6	7–19	20–34	0–6	7–19	20–34
	n = 208	n = 181	n = 35	n = 206	n = 173	n = 33	n = 181	n = 186	n = 42
Age, years, median (range)	36.0 (16–67)	38.0 (16–68)	38.0 (19–58)	36.0 (16–65)	40.0 (16–71)	37.0 (17–75)	38.0 (17–66)	36.0 (16–69)	38.5 (18–65)
Duration of epilepsy, years, mean (SD)	21.4 (14.4)	21.0 (13.6)	24.2 (11.9)	20.4 (12.7)	23.3 (12.9)	22.1 (11.8)	22.2 (13.0)	20.6 (12.6)	17.4 (10.6)
ASMs, n (%)									
1	56 (27.1)	54 (29.8)	6 (17.1)	62 (30.4)	37 (21.5)	7 (21.2)	53 (29.4)	53 (28.5)	11 (26.2)
2	143 (69.1)	123 (68.0)	29 (82.9)	137 (67.2)	129 (75.0)	24 (72.7)	122 (67.8)	127 (68.3)	30 (71.4)
≥3	8 (3.9)	4 (2.2)	0	5(2.5)	6 (3.5)	2 (6.1)	5 (2.8)	6 (3.2)	1 (2.4)
ASMs,^b^ n (%)									
Carbamazepine	96 (46.2)	82 (45.3)	19 (54.3)	107 (51.9)	77 (44.5)	19 (57.6)	97 (53.6)	87 (46.8)	20 (47.6)
Lamotrigine	54 (26.0)	47 (26.0)	7 (20.0)	44 (21.4)	44 (25.4)	3 (9.1)	45 (24.9)	48 (25.8)	11 (26.2)
Valproic acid	40 (19.2)	45 (24.9)	8 (22.9)	36 (17.5)	49 (28.3)	10 (30.3)	30 (16.6)	44 (23.7)	12 (28.6)
Levetiracetam	45 (21.6)	37 (20.4)	7 (20.0)	35 (17.0)	36 (20.8)	8 (24.2)	31 (17.1)	34 (18.3)	7 (16.7)
Use of any psychotropic medication,^c^ n (%)	13 (6.3)	17 (9.4)	4 (11.4)	22 (10.7)	20 (11.6)	6 (18.2)	12 (6.6)	20(10.8)	9 (21.4)
SSRI	7 (3.4)	11 (6.1)	0	14 (6.8)	14 (8.1)	3 (9.1)	7 (3.9)	12 (6.5)	4 (9.5)
Amitriptyline	3 (1.4)	1(0.6)	2(5.7)	3(1.5)	0	0	2(1.1)	4(2.2)	0
Citalopram	1(0.5)	2(1.1)	0	7(3.4)	4(2.3)	0	1(0.6)	4(2.2)	3(7.1)
Sertraline	0	3(1.7)	0	1(0.5)	6(3.5)	2(6.1)	3(1.7)	1(0.5)	0

OLE			
	OL ESL		
	Baseline MADRS total score		
	0–6	7–19	20–34
	n = 525	n = 507	n = 100
Age, years, median (range)	35.0 (16–70)	38.0 (16–71)	37.0 (17–75)
Duration of epilepsy, years, mean (SD)	20.9 (13.3)	21.7 (12.9)	20.9 (11.5)
ASMs, n (%)			
1	153 (29.2)	126 (24.9)	27 (27.0)
2	355 (67.7)	363 (71.7)	71 (71.0)
≥3	16 (3.1)	17 (3.4)	2 (2.0)
ASMs,^b^ n (%)			
Carbamazepine	267 (50.9)	221 (43.6)	56 (56.0)
Lamotrigine	125 (23.8)	136 (26.8)	20 (20.0)
Valproic acid	86 (16.4)	142 (28.0)	30 (30.0)
Levetiracetam	105 (20.0)	106 (20.9)	15 (15.0)
Use of any psychotropic medication,^c^ n (%)	42 (8.0)	52 (10.3)	21 (21.0)
SSRI	23 (4.4)	33 (6.5)	6 (6.0)
Medical history of psychiatric disorders, n (%)	92 (17.5)	121 (23.9)	28 (28.0)

(B)									
Controlled study period									
TEAE, n (%)	Placebo			ESL 800 mg			ESL 1200 mg		
	Baseline MADRS total score								
	0–6	7–19	20–34	0–6	7–19	20–34	0–6	7–19	20–34
	n = 204	n = 177	n = 42	n = 215	n = 164	n = 33	n = 182	n = 188	n = 38
Any psychiatric TEAE	10 (4.9)	16 (9.0)	6 (14.3)	7 (3.3)	21 (12.8)	4 (12.1)	8 (4.4)	18 (9.6)	3 (7.9)
	–	*P* = 0.1102^d^	***P* = 0.0247** ^**d**^	–	***P* = 0.0004** ^**d**^	***P* = 0.0213** ^**d**^	–	*P* = 0.0514^d^	*P* = 0.3680^d^
	–	–	–	–	*P* = 0.2640^e^	p = 0.7843^e^	–	*P* = 0.8605^e^	*P* = 0.3663^e^
Anxiety	3 (1.5)	4 (2.3)	2 (4.8)	0	8 (4.9)	1 (3.0)	0	1 (0.5)	0
Depression	1 (0.5)	4 (2.3)	2 (4.8)	0	5 (3.0)	1 (3.0)	2 (1.1)	6 (3.2)	1 (2.6)
Insomnia	3 (1.5)	0	0	3 (1.4)	4 (2.4)	1 (3.0)	2 (1.1)	2 (1.1)	1 (2.6)

OLE			
TEAE, n (%)	OL ESL		
	Baseline MADRS total score		
	0–6	7–19	20–34
	n = 525	n = 507	n = 100
Any psychiatric TEAE	44 (8.4)	58 (11.4)	15 (15.0)
	–	*P* = 0.0998^a^	***P* = 0.0380** ^**a**^
Anxiety	8 (1.5)	15 (3.0)	3 (3.0)
	–	*P* = 0.1185^a^	*P* = 0.3955^a^
Depression	6 (1.1)	11 (2.2)	7 (7.0)
	–	*P* = 0.1951^a^	***P* = 0.0017** ^**a**^
Insomnia	5 (1.0)	8 (1.6)	4 (4.0)

*Note:* Individual psychiatric TEAEs occurring in >1% of the total ESL population (and in >1 patient) are reported. For the controlled study period, the total ESL population included the 800 mg and 1200 mg doses, as well as a 400 mg dose group (data not reported here).

MADRS score categories: 0–6 = normal; 7–19 = mild depression; 20–34 = moderate depression.

*P*‐values <0.05 are in bold.

Abbreviations: ASM, anti‐seizure drug; ESL, eslicarbazepine acetate; MADRS, Montgomery–Åsberg Depression Rating Scale; OL, open‐label; OLE, open‐label extension; SD, standard deviation; SSRI, selective serotonin reuptake inhibitor; TEAE, treatment‐emergent adverse event.

^a^ Vs 0–6.

^b^ Psychiatric TEAEs are investigator‐reported Medical Dictionary for Regulatory Activities preferred terms and not clinical diagnoses.

^c^ Used by >15% of patients.

^d^ Used by >5% of patients.

^e^ Vs patients in the same MADRS score category in the placebo group.

**FIGURE 2:**
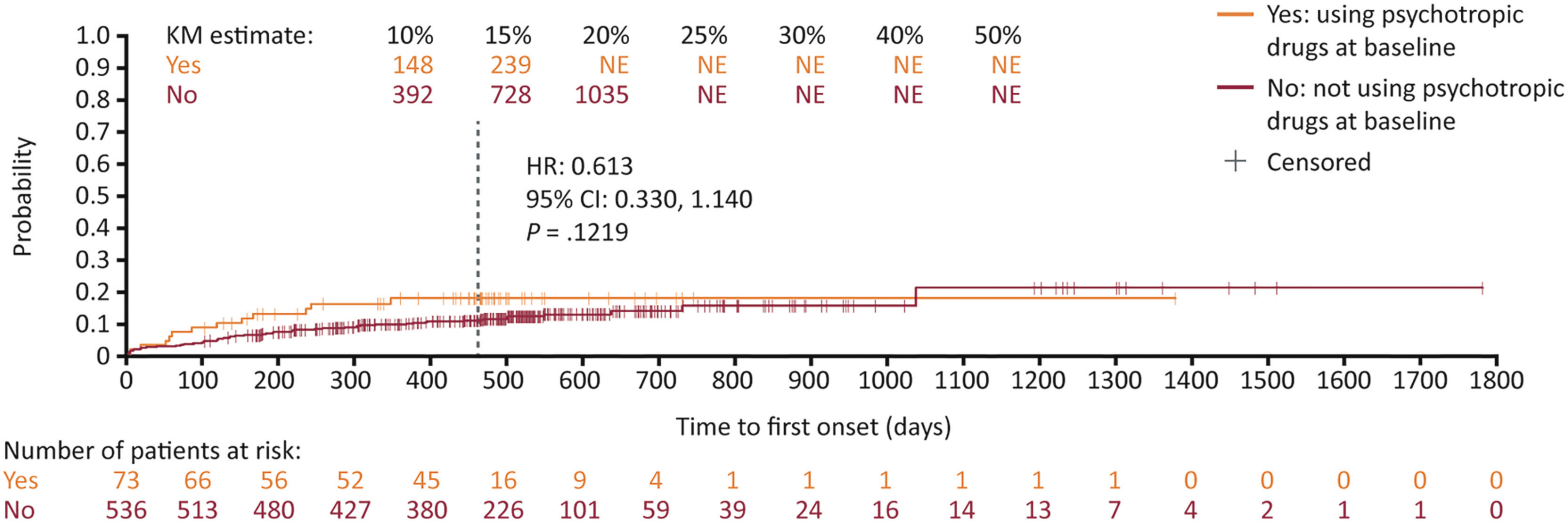
Time to first psychiatric TEAE during the controlled and 1‐year OLE study periods in patients with a MADRS score greater than 6. The vertical dashed line indicates the end of the 1‐year OLE. For patients who discontinued prior to completing the 1‐year OLE, their last contact date is reported as the censoring date. For patients who were ongoing at the completion of the 1‐year OLE, their end date was reported as the censoring date. Abbreviations: CI, confidence interval; HR, hazard ratio; KM, Kaplan–Meier; MADRS, Montgomery–Åsberg Depression Rating Scale; NE, not evaluable; OLE, open‐label extension; TEAE, treatment‐emergent adverse event

During the controlled study period, in the placebo group, the overall incidence of psychiatric TEAEs was higher in patients with moderate depression (MADRS total score 20–34) at baseline than in those categorized as “normal” (0–6) at baseline (Table 3B). In the ESL 800 mg group, the overall incidence of psychiatric TEAEs was higher in patients with mild (MADRS total score 7–19) or moderate depression at baseline, than in those categorized as “normal” at baseline. The relationship between baseline MADRS score and incidences of anxiety, depression, and insomnia were less clear. A Kaplan–Meier analysis of time to first psychiatric TEAE confirmed that psychiatric TEAEs occurred earlier and in a higher proportion of patients with moderate depression at baseline than in those with mild depression, or categorized as “normal” at baseline, during the controlled and 1‐year OLE study periods (7–19 vs 20–34: HR: 1.825 [95% CI: 1.03, 3.22]; *P* = 0.0383; 0–6 vs 20–34: HR: 1.546 [95% CI: 1.15, 2.08]; *P* = 0.0037) (Figure 1C). The difference was not statistically significant between patients categorized as “normal” or with mild depression at baseline (0–6 vs 7–19: HR: 1.300 [95% CI: 0.84, 2.01]; *P* = 0.2382) (Figure 1C). In patients with mild or moderate depression at baseline, there was no clear difference in psychiatric TEAE incidence between those taking ESL (either dose) and placebo (*P*‐values >0.05). A Kaplan–Meier analysis in a subset of patients with mild or moderate depression (MADRS >6) also found no difference in time to first psychiatric TEAE between patients taking or not taking psychotropic drugs during the controlled and 1‐year OLE study periods (HR: 0.613 [95% CI: 0.33, 1.14]; *P* = 0.1219; Figure 2).

During the OLE, the overall incidence of psychiatric TEAEs, as well as the incidence of depression, was higher in patients with MADRS scores indicative of moderate depression compared with patients categorized as “normal” during the OLE (Table 3B); odds ratios for “normal” vs moderate depression were 0.5 (0.3, 1.0) for the overall incidence of psychiatric TEAEs, and 0.2 (0.1, 0.5) for the incidence of depression.

## 4 DISCUSSION

In long‐term studies of adjunctive ESL in adults with focal seizures, there were no clear differences in frequencies of psychiatric events between patients taking ESL and placebo, and events were not clearly related to ESL dose. In controlled and OLE studies, psychiatric events generally occurred more frequently in patients with a medical history of psychiatric disorders, using psychotropic drugs, or with depressive symptoms than in those without a history, not using psychotropic drugs, or with no depressive symptoms.

Evaluation of baseline demographics and clinical characteristics identified some differences between patients with and without psychiatric symptoms/history, that is, between subgroups identified according to medical history of psychiatric disorders, baseline use of psychotropic drugs, or baseline MADRS total score. Patients with psychiatric symptoms/history were slightly older, consistent with reports that prevalence of depressive disorders increases with advancing age.^17^, ^18^ Additionally, although CBZ was the most frequently used ASM overall, a higher proportion of patients with psychiatric symptoms/history were taking LTG or LEV, and a higher proportion of patients without psychiatric symptoms/history were taking CBZ or VPA at baseline. All these ASMs have potential psychotropic properties, which could, at least in part, explain the uneven distribution between groups. Although we do not have detailed patient psychiatric histories or details of why individual patients were using specific concomitant ASMs, we can speculate regarding potential contributing factors for the uneven distribution across subgroups. LTG and LEV have putatively differing psychotropic properties, either of which could explain their relatively high use in patients with psychiatric symptoms/history. LTG has the potential to reduce depressive symptoms in patients with epilepsy, and thus may have been specifically chosen to simultaneously address seizures and depressive symptoms in patients who were experiencing psychiatric symptoms.^3^, ^18^, ^19^ Conversely, the higher use of LEV in patients with (vs without) current psychiatric symptoms or a psychiatric history is consistent with findings that use of LEV may be associated with an increased incidence of psychiatric side effects.^20^, ^21^ Although, LEV may also have potential in the treatment of psychiatric disorders.^22^ In addition, LTG or LEV might be preferable versus VPA or CBZ with regard to potential pharmacokinetic interactions with psychotropic drugs for the treatment of psychiatric disorders.^23^, ^24^, ^25^, ^26^ VPA and CBZ have mood‐stabilizing properties, which may be related to the relative absence of current or previous psychiatric symptoms in these subgroups.^19^, ^27^, ^28^, ^29^

There was a small amount of overlap across subgroups analyzed according to medical history of psychiatric disorders, baseline use of psychotropic drugs, or baseline MADRS score. Approximately 40% of patients with a psychiatric history were taking a psychotropic medication at baseline, and most patients (>80%) taking a psychotropic medication had a psychiatric history, as would be expected. The relatively low proportion of patients taking a psychotropic medication was likely due to the exclusion of those with a medically significant psychiatric history. Slightly higher MADRS scores were reported at baseline in patients with a psychiatric history or taking a psychotropic medication compared with those without a history or not taking a psychotropic medication. However, in patients with moderate depression (MADRS total score 20–34; OLE population), a psychiatric history was reported in fewer than 30% of patients and fewer than 25% of patients overall were taking a psychotropic medication. It is of note that patients with a reported medical history of psychiatric disorders in the current analysis did not have a medically significant psychiatric history or ongoing psychiatric illness (e.g., schizophrenia, bipolar disorder, major depressive disorder, suicide attempt, or other major psychiatric disorder), as patients with a history of major psychiatric disorders were excluded from the studies to avoid risk of destabilizing existing conditions.

Overall, the analyses found that patients with a medical history of psychiatric disorders, taking a psychotropic medication at baseline, or in the high MADRS total score category were generally more likely to experience a psychiatric TEAE than patients without psychiatric symptoms/history, both in the placebo and ESL treatment groups. The higher incidence of psychiatric TEAEs in patients with psychiatric symptoms/history was similar between the placebo and ESL treatment groups and was independent of ESL dose. The relationship between psychiatric history (but not baseline use of psychotropic drugs) and occurrence of psychiatric TEAEs was sustained over time, as demonstrated by Kaplan–Meier analyses. Differences between patients with and without psychiatric symptoms/history were less marked during the OLEs than during the controlled study periods; this could be due to the accumulating incidence of psychiatric events in patients without psychiatric symptoms/history, as would be expected during monitoring over a long period of time (1 year), or due to the discontinuation of patients with significant tolerability issues removing them from the analysis population.

The results are consistent with previous findings that in patients with epilepsy, a history of psychiatric disorders can be a risk factor for experiencing a new psychiatric disorder.^2^, ^3^, ^4^, ^16^ In addition, we were unable to establish from the current analysis whether the occurrence of psychiatric TEAEs in patients taking psychotropic medications at baseline was a consequence of the underlying symptoms that necessitated taking the medication (e.g., a psychiatric comorbidity), related to the increased risk associated with a psychiatric history, or due to other factors that differed between subgroups; whatever the cause, the differences between patients taking/not taking psychotropic medications at baseline decreased over time.

Depression and anxiety were the most frequently reported psychiatric TEAEs and appeared to be primarily responsible for the higher incidence of psychiatric TEAEs in patients with psychiatric symptoms/history compared with those without psychiatric symptoms/history. The incidence of depression during the controlled study period in patients using versus not using psychotropic drugs at baseline was high in the ESL 1200 mg group. However, this is unlikely to indicate that the occurrence of depression was related to ESL dose, as in patients taking psychotropic medications at baseline, the differences in incidences of depression between those taking ESL 1200 mg (16%) or ESL 800 mg (8%) vs placebo (9%) were not significant (*P* = 0.4245 and *P* = 0.7665). Insomnia, depressed mood, and nightmare were psychiatric TEAEs that also occurred in >1% and >1 patient in the total ESL group in some of the analyzed subgroups during the controlled study period, and aggression and confusional state were additional psychiatric TEAEs detected during the OLEs. During the OLEs, there was no placebo group to determine whether rates of psychiatric TEAEs were elevated above expected levels. A larger dataset would be required to evaluate the relationship between psychiatric history/current psychiatric status and the less frequently occurring psychiatric disorders.

Limitations of the analysis include the post–hoc nature, small subgroup sizes (particularly during the controlled studies), the relatively short duration of the controlled study period, and differences in baseline ASM use and age across subgroups, which could affect likelihood of drug–drug interactions, potential psychotropic effects related to specific ASMs, and vulnerability to TEAEs. However, the general trends are clear, particularly during the OLE, and consolidate previous analyses with these new data from three Phase III clinical trials. Although patients with a history of major psychiatric disorders were excluded from the studies, five patients had baseline MADRS scores >34, suggesting presence of severe depressive symptoms. These patients did not meet exclusion criteria, as the MADRS was used for safety analyses during the studies and not as a screening instrument. The studies did not use prospective tools to assess psychiatric diagnoses; patients were categorized according to medical history of psychiatric disorders based on questioning during screening. A potential confounder was that categorization according to baseline use of psychotropic drugs did not consider ASMs with potential psychotropic effects; it was not possible to further evaluate this limitation, as specific psychotropic effects of ASMs in individual patients are unknown. In addition, as psychotropic drug use was evaluated at baseline only, it is not known whether use changed throughout the duration of the study. Another limitation was that all psychiatric TEAEs were based on TEAE reporting alone and were not confirmed by formal testing to provide evidence that incident psychiatric symptoms represented a clinical diagnosis. *P*‐values were calculated without correcting for multiplicity or the post–hoc nature of the hypotheses, and so the results should be interpreted with caution.

## 5 CONCLUSIONS

Overall, in clinical trials of ESL in adults with focal seizures, there was no clear difference in the incidence of psychiatric AEs between patients taking placebo and ESL. Psychiatric events generally occurred more frequently in patients with baseline psychiatric symptoms or a history of psychiatric disorders. Long‐term exposure to ESL was not associated with a marked increase in the incidence of psychiatric TEAEs.

## FUNDING INFORMATION

Sunovion Pharmaceuticals Inc., Marlborough, Massachusetts, USA, and BIAL – Portela & C^a^, S.A., São Mamede do Coronado, Portugal funded the clinical trials reported in this manuscript. The sponsors were involved in the study design, in the collection, analysis and interpretation of data, in the writing of the report, and in the decision to submit the article for publication.

## CONFLICT OF INTEREST

Hamada Altalib: consultant/advisory board for Eisai; research grant from VA Healthcare System and US Department of Defense CDMRP Epilepsy Research Program; other research support from Sunovion Pharmaceuticals Inc., UCB, Engage, Eisai, and Epilepsy Consortium. Todd Grinnell, David Cantu, and Yi Zhang: employees of Sunovion Pharmaceuticals Inc. Fábio Ikedo and Mariana Vieira: employees of BIAL – Portela & Cª, S.A. David Blum: paid consultant to Sunovion Pharmaceuticals Inc. Note that Sunovion Pharmaceuticals Inc acquired the rights to eslicarbazepine acetate in the United States and Canada markets under an exclusive license from BIAL– Portela & Cª.

## ETHICAL APPROVAL

We confirm that we have read the Journal's position on issues involved in ethical publication and affirm that this report is consistent with those guidelines.

## Data Availability

Sunovion Pharmaceuticals Inc. is part of a clinical trial data‐sharing consortium that facilitates access for qualified researchers to selected anonymized clinical trial data. For up‐to‐date information on data availability, please visit: https://www.clinicalstudydatarequest.com/Study‐Sponsors.aspx and click on Sunovion.
